# Structure Bioinformatics of Six Human Integral Transmembrane Enzymes and their AlphaFold3 Predicted Water-Soluble QTY Analogs: Insights into FACE1 and STEA4 Binding Mechanisms

**DOI:** 10.1007/s11095-025-03822-6

**Published:** 2025-02-18

**Authors:** Edward Chen, Emily Pan, Shuguang Zhang

**Affiliations:** 1 Carnegie Mellon University, Pittsburgh, PA USA; 2 Massachusetts Institute of Technology, 77 Massachusetts Avenue, Cambridge, MA 02139 USA; 3 Media Lab, Massachusetts Institute of Technology, Cambridge, MA 02139 USA

**Keywords:** bioinformatics, biophysics proteins, computational biophysics, membrane protein structure, protein dynamics simulation

## Abstract

**Objective:**

Human integral membrane enzymes are essential for catalyzing a wide range of biochemical reactions and regulating key cellular processes. However, studying these enzymes remains challenging due to their hydrophobic nature, which necessitates the use of detergents. This study explores whether applying the QTY code can reduce the hydrophobicity of these enzymes while preserving their structures and functions, thus facilitating bioinformatics analysis of six key integral membrane enzymes: MGST2, LTC4S, PTGES, FACE1, STEA4, and SCD.

**Methods:**

The water-soluble QTY analogs of the six membrane enzymes were predicted using AlphaFold3. The predicted structures were superposed with CyroEM determined native structures in PyMOL to observe changes in structure and protein-ligand binding ability.

**Results:**

The native membrane enzymes superposed well with their respective QTY analogs, with the root mean square deviation (RMSD) ranging from 0.273 Å to 0.875 Å. Surface hydrophobic patches on the QTY analogs were significantly reduced. Importantly, the protein-ligand interactions in FACE1 and STEA4 were largely preserved, indicating maintained functionality.

**Conclusion:**

Our structural bioinformatics studies using the QTY code and AlphaFold3 not only provide the opportunities of designing more water-soluble integral membrane enzymes, but also use these water-soluble QTY analogs as antigens for therapeutic monoclonal antibody discovery to specifically target the key integral membrane enzymes.

## Introduction

There are a very large number of integral membrane enzymes in all living systems that are vital for diverse biological process. But they are not well studied due to the significant challenges to obtain large quantities to carry out detailed studies. Thus, we here carry out the structural bioinformatic studies.

We focus on six proteins across four distinct families: the MAPEG (Membrane-Associated Proteins in Eicosanoid and Glutathione metabolism) family, the STEAP (Six-transmembrane epithelial antigen of prostate) family, the peptidase M48A family, and the fatty acid desaturase type 1 family. Despite belonging to different families, these proteins share critical roles in lipid metabolism and catalyzing essential biochemical reactions. Each family contributes uniquely to cellular processes, from the MAPEG family's involvement in eicosanoid and glutathione metabolism [1, 2] to the STEAP family's role in metal ion reduction and transport [3]. The peptidase M48A family is crucial in protein processing, and the fatty acid desaturase type 1 family is integral in lipid modification, demonstrating the diverse yet interconnected functions of these proteins in maintaining cellular homeostasis.

The MAPEG family is characterized by its involvement in eicosanoid and glutathione metabolism, playing key roles in i) glutathione S-transferase (GST) catalysis, ii) the synthesis of inflammatory mediators such as leukotrienes and prostaglandins, iii) the detoxification of reactive oxygen species and harmful xenobiotics, and iv) the regulation of oxidative stress and cell survival pathways [4]. These functions make MAPEG proteins indispensable in processes ranging from inflammation and immune response to cellular protection and tumor development. The MAPEG superfamily consists of 136 known proteins, six of which are found in humans [4, 5]. These proteins exist as homotrimers with the active site residing at subunit interfaces [5, 6]. The conjugation of GSH to substrate occurs within the hydrophobic binding pocket, as indicated by the similar hydropathy profiles of the MAPEG proteins, especially in the alignment positions 80–120 [1, 6]. Hydropathy plots further suggest at least three membrane-spanning segments, with a possible fourth yet to be experimentally confirmed [5]. The MAPEG family are linked to various human diseases, including a) asthma, b) cancer, c) inflammatory diseases, d) neurological diseases, and more.

MGST2, a member of the MAPEG family, is primarily found in the liver, lung, and heart [2]. It plays a crucial role in producing leukotriene C4, a molecule essential for intracrine signaling related to ER stress, oxidative DNA damage, and cell death [2, 7, 8]. Due to these functions, MGST2 is a significant target in drug development, particularly in efforts to generate LTC_4_ that can mediate ER stress and chemotherapy triggered oxidative stress and oxidative DNA damage. Inhibition targeting MGST2 could significantly reduce the oxidative stress and DNA damage induced by chemotherapy and ER stress. This inhibition leads to a significant reduction in cell death mouse morbidity, suggesting that MGST2-generated LTC_4_ plays a pivotal role in mediating the harmful effects of ER stress and chemotherapy [7].

LTC4S, a member of the MAPEG family, is expressed in the lungs, platelets, and the myelogenous leukemia cell line KG-1. This enzyme plays a pivotal role in catalyzing the production of leukotriene C4 by conjugating glutathione (GSH) with leukotriene A4 (LTA4) [9, 10]. Research has shown that LTC4S is overexpressed in individuals with an aspirin-induced asthmatic phenotype, leading to the overproduction of cysteinyl leukotrienes, increased bronchial hyperreactivity to lysine aspirin, and enhanced peptide leukotriene biosynthesis [11, 12]. Currently, Zileuton serves as a 5-lipoxygenase (5-LO) inhibitor, to prevent the production of leukotrienes in the pathophysiology of asthma [13]. Drug design targeting LTC4S could serve a similar function in inhibiting the production of leukotriene.

PTGES, a key mediator in the inflammatory response, has emerged as an important drug target for the treatment of pain [14, 15]. Structural analysis of PTGES reveals a small, well-defined domain between helices I and II, which leads to the active site cavity [15]. This structural feature offers potential opportunities for the design of mPGES-1 inhibitors, making PTGES a promising target for therapeutic intervention [14, 15]. Compared to previous efforts in inhibiting PGE_2_ formation, which mitigates inflammation at the risk of cardiovascular adverse events due to suppression of antithrombotic prostacyclin production, inhibition of mPGES-1 targets the prostaglandin pathway downstream of PGH2 synthesis and circumvents the side effects [16].

MGST2, LTC4S, and PTGES are all members of the membrane-associated proteins in the eicosanoid and glutathione metabolism (MAPEG) family. MGST2 is very similar to LTC4S, with the two proteins sharing 44% similarity in their sequence [17]. However, MGST2 differs from LTC4S in that it displays reactivity in only one-third of the sites to activate its substrate GSH [17, 18]. PTGES is more similar to MGST1, sharing a 39% sequence identity.

FACE1 (Farnesylated-proteins converting enzyme 1), which is also called ZMPSTE24, is a member of the peptidase M48A family. FACE1 is crucial for the proteolytic cleavage of prelamin A at the CaaX motif at its C-terminal end, as well as a secondary cleavage site located 15 residues upstream from the CaaX site [19]. Mutations in FACE1 disrupt the processing of prelamin A, often leading to premature aging diseases such as Hutchinson-Gilford progeria syndrome (HGPS) [20–23]. It was also observed that HIV protease inhibitors used to treat HIV infections inhibits FACE1 as an off-target effect, hindering the normal processing of prelamin A [19]. In addition, FACE1 restricts RNA and DNA viruses including influenza A, Ebola, Zika, and cowpox by forming complex with interferon-induced transmembrane protein (IFITM) family proteins to impede viral entry [21].

The STEAP family is characterized by its inherent metalloreductase activity, playing vital roles in i) metal metabolism, ii) molecular trafficking in the endocytic and exocytic pathways, and iii) regulation of cell proliferation and apoptosis [3]. These proteins are characterized by a six-transmembrane helix structure with intracellular N- and C-terminal domains, facilitating ion channeling and protein transport [24]. Overexpression of STEAP family proteins occur in several types of human cancer, such as a) prostate, b) bladder, d) colon, d) pancreas, e) ovary and testis, and more, making them potential therapeutic targets for cancer treatment [3].

STEA4 is a member of the STEAP family, which is primarily involved in the reduction of Fe^3^⁺ (ferric) and Cu^2^⁺ (cupric) ions, making these metals bioavailable for various cellular processes [25]. STEA4 is notably upregulated in prostate, colon, and bladder cancers, suggesting that targeting STEA4 could disrupt the metabolic needs of cancer cells and potentially inhibit their growth [25, 26]. Additionally, STEA4 is closely associated with metabolic disorders such as obesity and Type 2 diabetes [26, 27]. Pharmacological studies have shown that blockage of the STEA4 pathway with an iron chelator (deferiprone) with Lanatinib to inhibit HER2 significantly reduced breast cancer cell growth. siRNA mediated lockdown of STEA4 also suppressed cell proliferation and overexpression of HER2 in breast cancer, showing STEA4 as a critical drug target for breast cancer [28].

SCD (Stearoyl-CoA desaturase) is a member of the fatty acid desaturase type 1 family. It is crucial in lipid metabolism by generating monounsaturated fatty acids associated with cell growth, survival, and metabolic regulation [29–31]. The over-expression of SCD has been linked to various metabolic diseases, highlighting its potential as a therapeutic target. Inhibitors of SCD are thus considered promising candidates for treating metabolic disorders and cancer [29–31]. SCD inhibition has been targeted at epithelial ovarian cancer (EOC) to significantly reduce the proliferation of EOC cells and patient-derived organoids and induced apoptotic cell death, while not affecting the non-cancer cells [31].

Traditionally, researchers have used methods such as X-ray crystallography and NMR spectroscopy to determine protein structures. Recently, CryoEM (Cryo-electron microscopy) has emerged as the predominant technique for studying cellular architecture [32, 33]. This method involves imaging frozen specimens maintained at temperatures of liquid nitrogen or nitrogen helium, allowing for high-resolution structural analysis [34].

In our study, we focus on proteins for which CryoEM structures are available with high resolution, ranging from 1.16 Å to 3.25 Å. Despite these advancements, studying the structure and function of multi-segment transmembrane proteins still remains challenging. These proteins require detergents for solubilization after extraction from cell membranes, which complicates and prolongs the process of structural elucidation [35].

To overcome the difficulties of studying membrane proteins, we used the simple QTY code to engineering water-soluble membrane proteins based on the 1.5 Å electron density map, which shows structural similarities between leucine (L) *vs* glutamine (Q); isoleucine (I)/valine (V) *vs* threonine (T); and phenylalanine (F) *vs* tyrosine (Y) [36–38]. Applying the simple QTY code previously, we bioengineered several detergent-free chemokine receptors and cytokine receptors. These water-soluble QTY analogs were expressed and purified, they are shown to be not only structurally stable but they also retained their ligand-binding capability [36–40].

After the advent AlphaFold2 became available in 2021, we used AlphaFold2 to predict membrane protein QTY analog protein structures. The QTY code was first applied to 7 chemokine receptors [41], glucose transporters [42], solute carrier transporters [43], ABC transporters [44], and neurological transporters such as serotonin, norepinephrine, dopamine transporters [45] and another synaptic vesicle protein subgroup of glutamate transporters (VGLUTs) [46]. We also designed reverse QTY analogs of Human Serum Albumin to effectively facilitate the release of anti-tumor drugs in mice [47]. The water-soluble chemokine receptor CXCR4^QTY^ analog has been successfully used in biomimetic sensors [48].

In May 2024, AlphaFold was upgraded to version 3, namely AlphaFold3, featuring an enhanced diffusion-based architecture capable of accurately predicting the joint structures of protein complexes and extending its capabilities beyond protein predictions to include DNA, RNA, and small molecules such as ligands [49, 50].

To build on top of our previous studies and utilize AlphaFold3’s advance capabilities, we are now studying the protein-protein and protein-ligand interactions of two key integral membrane enzymes: FACE1 and STEA4. FACE1 is essential for the proteolytic processing of prelamin A. FACE1's active site is housed within a closed chamber formed by seven transmembrane spans, with side portals allowing substrate entry. To date, prelamin A is the only known substrate of FACE1, and improper processing of prelamin A can lead to premature aging diseases [22, 23]. STEA4 reduce Fe^3+^and Cu^2+^ ions to facilitate metal-ion uptake by mammalian cells and the overexpression of STEA4 is linked to several different types of cancer [25]. STEAP4 has a trimeric structure that aligns NADPH with FAD and a b-type heme across the membrane, facilitating electron transfer [25]. This structure enables substrate binding and the reduction of iron, which associates with a positively charged ring and its chelator.

Thus, we used AlphaFold3 to test the structural stability of QTY analogs. In addition, we conducted bioinformatic studies using AlphaFold3 to predict the substrate binding ability of QTY analogs compared to their native structures. Here, we report the structural bioinformatic studies of six experimentally determined proteins and their AlphaFold3-predicted water-soluble QTY analogs. We also provide the superpositions of native and QTY analog proteins, their surface hydrophobicity analyses, and finally the substrate binding analyses of the hydrophobic proteins and their hydrophilic QTY analogs.

## Results and Discussion

### The Rationale of the QTY Code

**Fig. 1:**
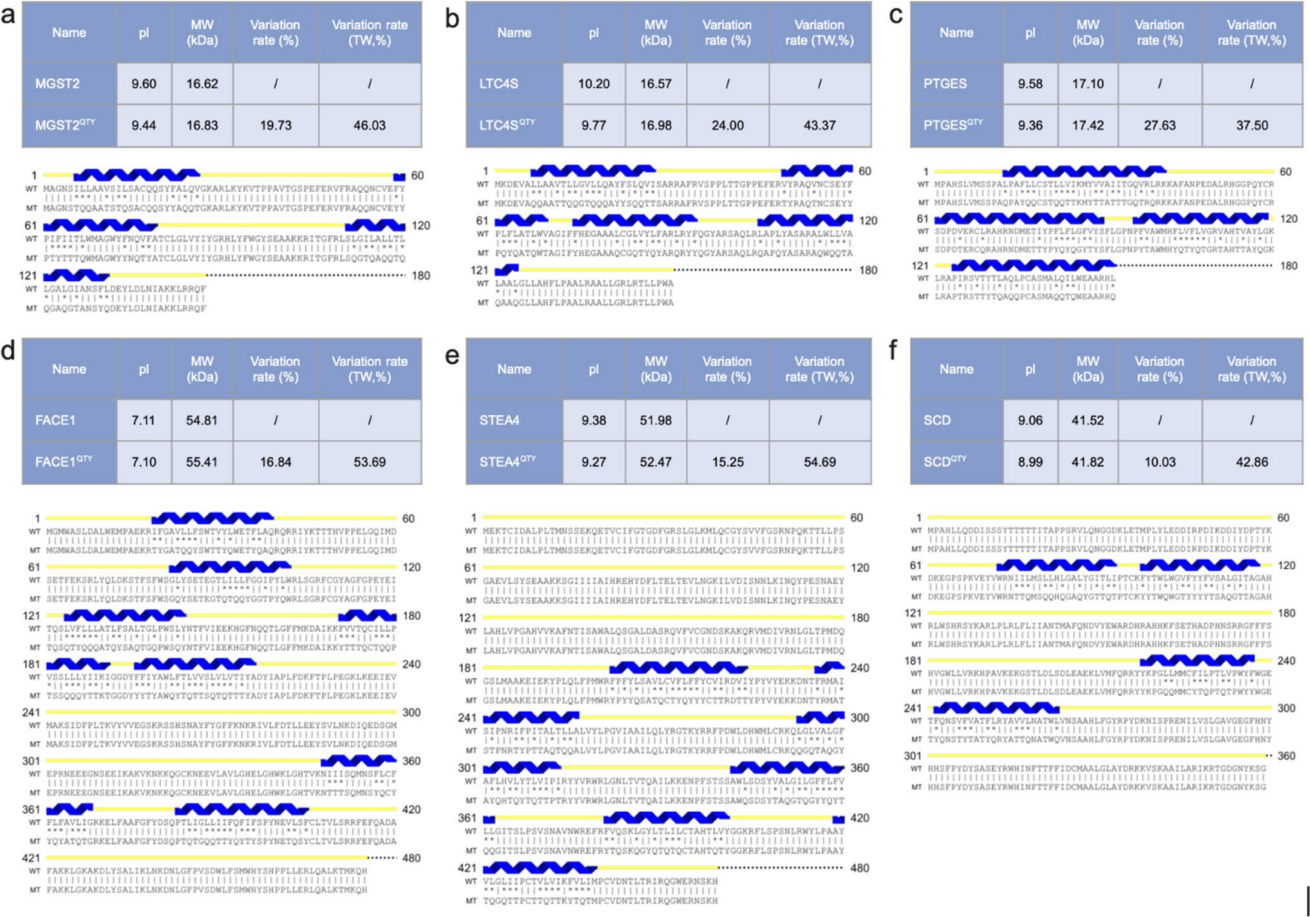
Protein sequence alignments of six integral membrane enzymes with their water-soluble QTY analogs. The symbols | and * indicate whether amino acids are identical or different, respectively. Please note the Q, T, and Y amino acids (red) replacing L, V and I, and F, respectively. The alpha helices (blue) are shown above the protein sequences. The characteristics of natural and QTY analogs listed are isoelectric focusing (pI), molecular weight (MW), total variation %, and transmembrane variation %. The alignments are: (**a**) MGST2 *vs* MGST2^QTY^, (**b**) LTC4S *vs* LTC4S^QTY^, (**c**) PTGES *vs* PTGES^QTY^, (**d**) FACE1 *vs* FACE1^QTY^, (**e**) STEA4 *vs* STEA4^QTY^, and (**f**) SCD *vs* SCD^QTY^. Although there are significant QTY changes in the TM alpha helices (37.50–54.69%), their changes in MW and pI are insignificant. (Please see Figure S1 for more enlarged protein sequence alignments for clarity).

The hydrophobic nature of integral transmembrane enzymes presents significant challenges to studying their structure and function. The striking structural similarities between the electronic density maps of Q and L, T and V/I, and Y and F stimulated us to ask the question if these amino acids can be interchanged. Thus, the pairwise QTY replacements were applied as a systematic way to reduce the hydrophobicity of integral transmembrane enzymes [36, 37]. The QTY code can simply enable engineering of transmembrane enzymes into their water-soluble analogs by replacing hydrophobic amino acids with hydrophilic ones: leucine (L) with glutamine (Q), isoleucine (I) and valine (V) with threonine (T), and phenylalanine (F) with tyrosine (Y). The QTY substitutions effectively reduce the hydrophobic surfaces on these transmembrane enzymes. Despite substantial changes in protein sequences and amino acid composition in the transmembrane domain of these native transmembrane enzymes, their QTY analogs exhibit similar isoelectric points (pI) and molecular weights (MW) (Fig. 1).

### Protein Sequence Alignments and other Characteristics

The protein sequences of the six transmembrane enzymes are aligned with their QTY analogs (Fig. 1). The changes made for the QTY analog are highlighted by the “*” symbol, leading to an overall change of amino acid composition from 10.03% to 27.63% and changes in the transmembrane domains from 37.50% to 54.69% (Fig. 1, Table I). However, the isoelectric point (pI) remained similar because Q (glutamine), T (threonine), and Y (tyrosine) are neutral without any charges, hence, their substitution does not introduce acidic or basic amino acids. The molecular weight (MW) only has a slight increase, as leucine (L: 131.17 Da) is lighter than glutamine (Q: 146.14 Da), isoleucine (I: 131.17 Da) and valine (V: 117.15 Da) are both lighter than threonine (T: 119.12 Da), and phenylalanine (F: 165.19 Da) is lighter than tyrosine (Y: 181.19 Da). On a more fundamental basis, the substitution result in original carbon side chains (12 Da) to be replaced by side chains containing oxygen (-OH, 16 Da) and nitrogen (-NH_2_, 14 Da).

**Table I:**
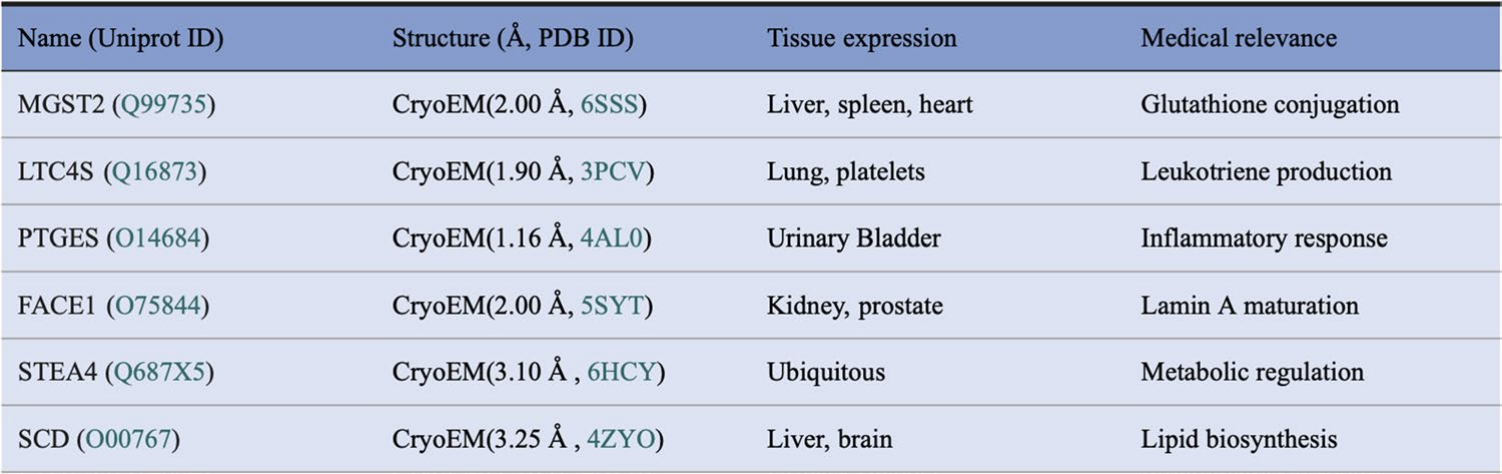
The Protein Names, UniProt ID, and CryoEM Structure (Å) with PBD ID

The lists of tissue location, medical relevance, and function are not exhaustive. Updated results become available from more and more recent studies

### Accuracy of AlphaFold3 Protein Structure Predictions

There are several metrics used to assess the accuracy of structural predictions made by AlphaFold3, including the Predicted Local Distance Difference Test (pLDDT)**,** Predicted Template Modeling score (pTM), and Predicted Aligned Error (PAE).

**Fig. 2:**
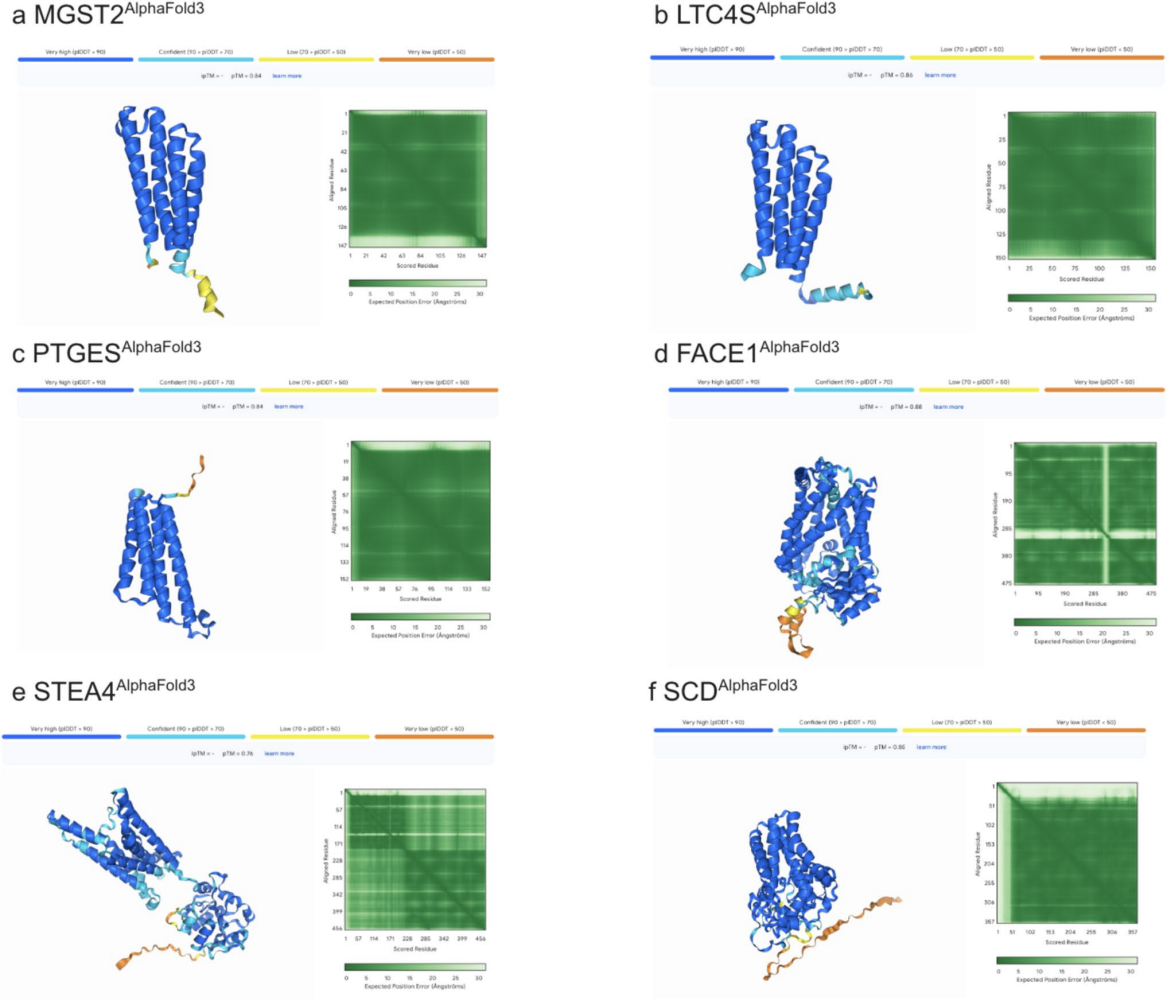
The pLDDT and AlphaFold 3 predicted integral membrane enzyme water-soluble QTY analogs. The pLDDT confidence interval, pTM score, and PAE matrix for the six predicted human integral membrane enzymes. The transmembrane regions are predicted well with high accuracy (dark blue). (**a**) MGST2^QTY^, (**b**) LTC4S^QTY^, (**c**) PTGES^QTY^, (**d**) FACE1^QTY^, (**e**) STEA4^QTY^, and (**f**) SCD^QTY^. The unstructured parts are predicted with less certainty (orange and yellow colors).

The pLDDT measures the confidence in the positioning of each individual residue in the predicted structure [51]. In AlphaFold3 predictions, pLDDT values are color-coded to represent confidence: dark blue regions (pLDDT > 90) indicate very high confidence and are expected to be modeled with high accuracy, light blue regions (pLDDT between 70 and 90) are expected to be modeled well, yellow (pLDDT between 50 and 70) indicates moderate confidence, and orange (pLDDT < 50) suggests low confidence in the accuracy of those regions [52]. Our predictions show that transmembrane regions are modeled with high accuracy, consistent with the expected behavior for well-structured protein regions (Fig. 2). However, the regions at the ends of the structures are predicted with lower confidence, indicating these are intrinsically disordered or unstructured [53]. These regions often display a ribbon-like appearance in the structural model. In our study, we chose to exclude these low-confidence, unstructured loops from the final models to focus on the more reliable, structured regions.

pTM evaluates the overall quality of the protein structure by comparing it to known templates [54]. This score provides an indication of how reliable the overall prediction is. In our study, the predicted structures achieved an average pTM score of 0.84 (Fig. 2), demonstrating that the models are highly accurate and closely resemble experimentally solved structures.

PAE extends the concept of pLDDT by measuring the predicted distance error between pairs of residues in the structure [53]. This is often displayed as a symmetric matrix, where the entry at row i and column j shows the predicted error between residues i and j. The diagonal elements represent the individual residue's predicted error. A low PAE value (e.g., dark green) between two residues suggests that AlphaFold is confident in their relative spatial arrangement. In our predictions, we observe that the matrix is largely shaded in dark green, indicating high confidence in the structural model for most residues (Fig. 2).

Overall, the AlphaFold3 predictions indicate a high level of accuracy based on the available metrics (Fig. 2). However, it is important to remember that these are still predictions and should not be treated as definitive "ground truth." While these predictions are valuable for exploring the potential engineering of QTY analogs of various enzymes, further experimental validation in the laboratory will be necessary to confirm these findings.

### Superpositions of Native CryoEM Transmembrane Enzymes and their Water-Soluble QTY Analogs

**Fig. 3:**
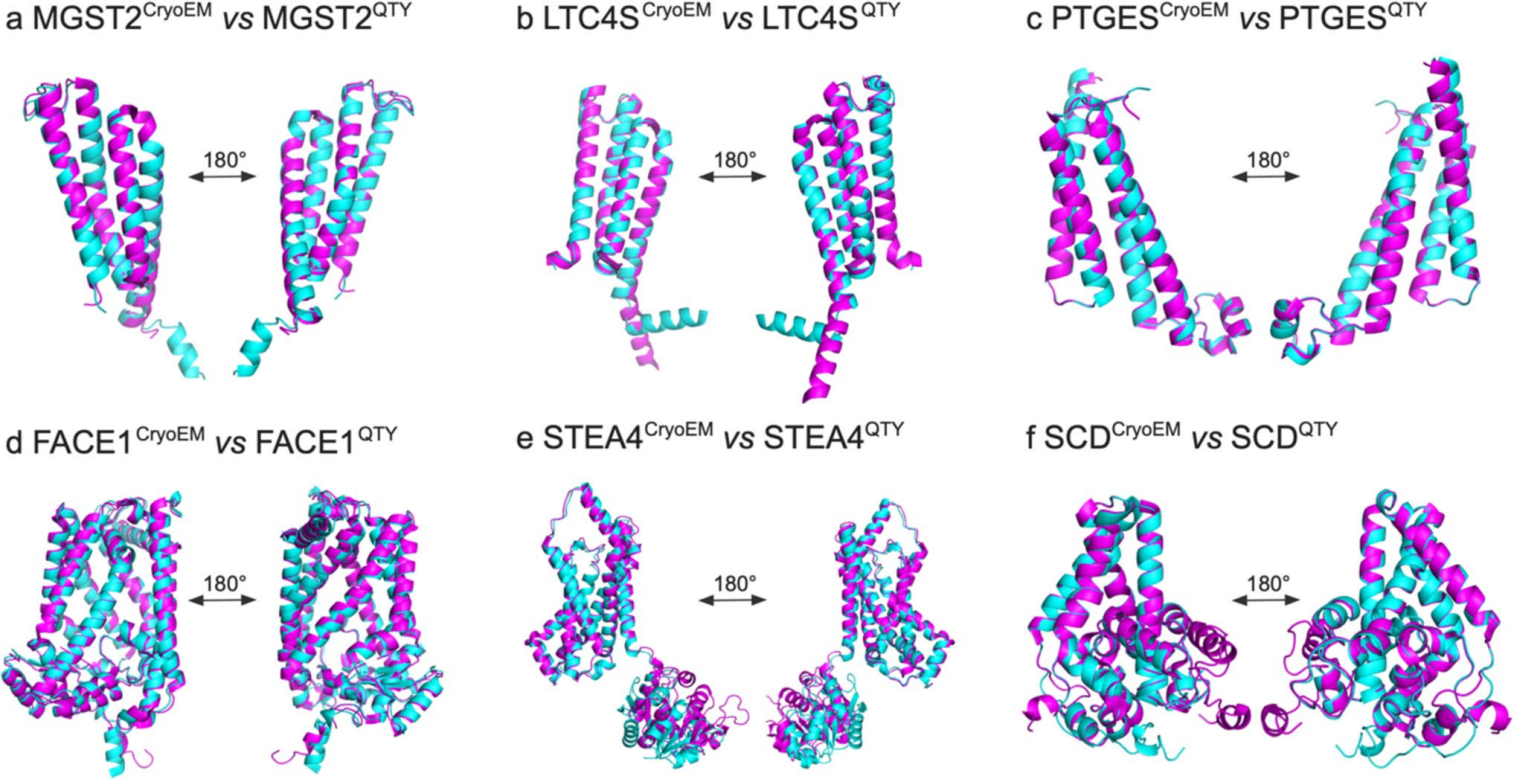
Superpositions of six human Cryo-EM-determined structures of integral membrane enzymes and their AlphaFold3-predicted water-soluble QTY analogs. The CryoEM determined structures of the native transporters are obtained from the Protein Data Bank (PDB). The CryoEM structures (magenta) are superposed with their QTY analogs (cyan) predicted by AlphaFold3. These superposed structures show that the membrane proteins and their QTY analogs have very similar structures. For clarity of direct comparisons, unstructured loops in the CryoEM structures were removed in the QTY analogs. (**a**) MGST2 *vs* MGST2^QTY^, (**b**) LTC4S *vs* LTC4S^QTY^, (**c**) PTGES *vs* PTGES^QTY^, (**d**) FACE1 *vs* FACE1^QTY^, (**e**) STEA4 *vs* STEA4^QTY^, and (**f**) SCD *vs* SCD^QTY^.

We ask if the molecular structure of six membrane enzymes can be well superposed with their respective QTY analogs (Fig. 3). The native structures of MGST2 (PDB:6SSS) [8], LTC4S (PDB: 3PCV) [55], PTGES (PDB: 4AL0) [15], FACE1 (PDB: 5SYT) [19], STEA4 (PDB: 6HCY) [25], and SCD (PDB: 4ZYO) [29] have been experimentally determined through CryoEM and the QTY analogs are predicted through AlphaFold3. The superpositions of the transmembrane enzymes and their respective QTY analogs are: MGST2 *vs* MGST2^QTY^, LTC4S *vs* LTC4S^QTY^, PTGES *vs* PTGES^QTY^, FACE1 *vs* FACE1^QTY^, STEA4 *vs* STEA4^QTY^, SCD *vs* SCD^QTY^ (Fig. 3).

The native membrane enzymes superposed well with their respective QTY analogs, with the root mean square deviation (RMSD) ranging from 0.273 Å to 0.875 Å (Table II). These superpositions not only demonstrate the high accuracy of the AlphaFold3 server in protein structure prediction, but also suggest that the structural changes between the native proteins and their QTY analogs are minimal despite substantial replacements in their amino acid sequences.

**Table II:**
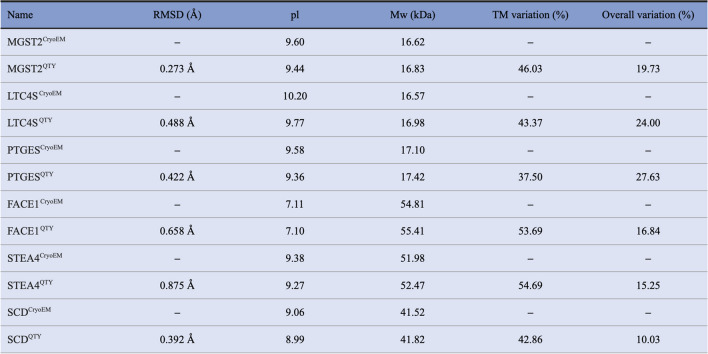
The Characteristics of Integral Membrane Protein Enzymes and their QTY Variants

pI, isoelectric point; MW, molecular weight; TM, transmembrane; –, not applicable, and RMSD, residue mean square distance. The six multi-pass membrane proteins are listed in the same order as Fig. 1. RMSDs were calculated after missing residuals (unstructured loops) in the native CryoEM-determined structures and the corresponding residuals in the predicted QTY structures were cut out. If the native protein was a dimer, one monomer was also cut out. The QTY amino acid substitutions in the transmembrane (TM) are significant between 37.50% and 54.69%, whereas the overall structural changes are between 10.03% and 27.63%

### Superpositions of AlphaFold3-Predicted Native Transmembrane Enzymes and their Water-Soluble QTY Analogs

**Fig. 4:**
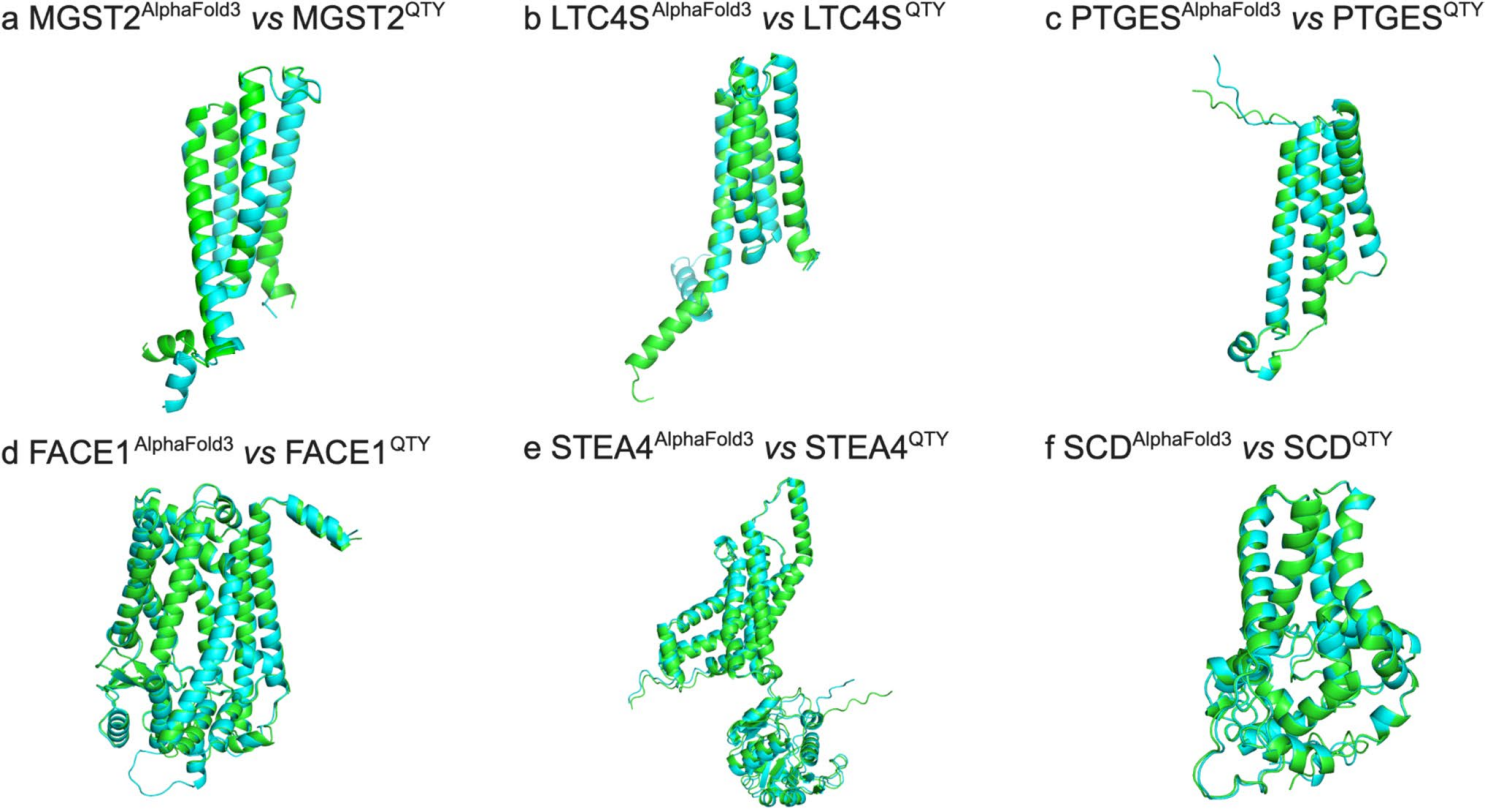
Superpositions of AlphaFold3-predicted structures of native and their QTY enzyme analogs. Color code: green = AlphaFold3-predicted native structures; cyan = AlphaFold3-predicted water-soluble QTY analogs. (**a**) MGST2 *vs* MGST2^QTY^ (RMSD = 0.273 Å), (**b**) LTC4S *vs* LTC4S^QTY^ (RMSD = 0.488 Å), (**c**) PTGES *vs* PTGES^QTY^ (RMSD = 0.422 Å), (**d**) FACE1 *vs* FACE1^QTY^ (RMSD = 0.658 Å), (**e**) STEA4 *vs* STEA4^QTY^ (RMSD = 0.875 Å), and (**f**) SCD *vs* SCD^QTY^ (RMSD = 0.392 Å).

We ask how well the AlphaFold3-predicted native transmembrane enzymes superpose with their QTY analogs, we superposed the AlphaFold3-predicted native structures with their water-soluble QTY analogs (Fig. 4). The structures superposed very well with low RMSD: a) MGST2 *vs* MGST2^QTY^ (RMSD = 0.247 Å), b) LTC4S *vs* LTC4S^QTY^ (RMSD = 0.453 Å), c) PTGES *vs* PTGES^QTY^ (RMSD = 0.420 Å), d) FACE1 *vs* FACE1^QTY^ (RMSD = 0.410 Å), d) STEA4 *vs* STEA4^QTY^ (RMSD = 0.920 Å), f) SCD *vs* SCD^QTY^ (RMSD = 0.251 Å). The superposition with low RMSD shows that the water-soluble QTY analogs share very similar structures with the native transmembrane enzymes.

### Superpositions of CryoEM Structures with AlphaFold3-Predicted Native Transmembrane Enzymes and their Water-Soluble QTY Analogs

**Fig. 5:**
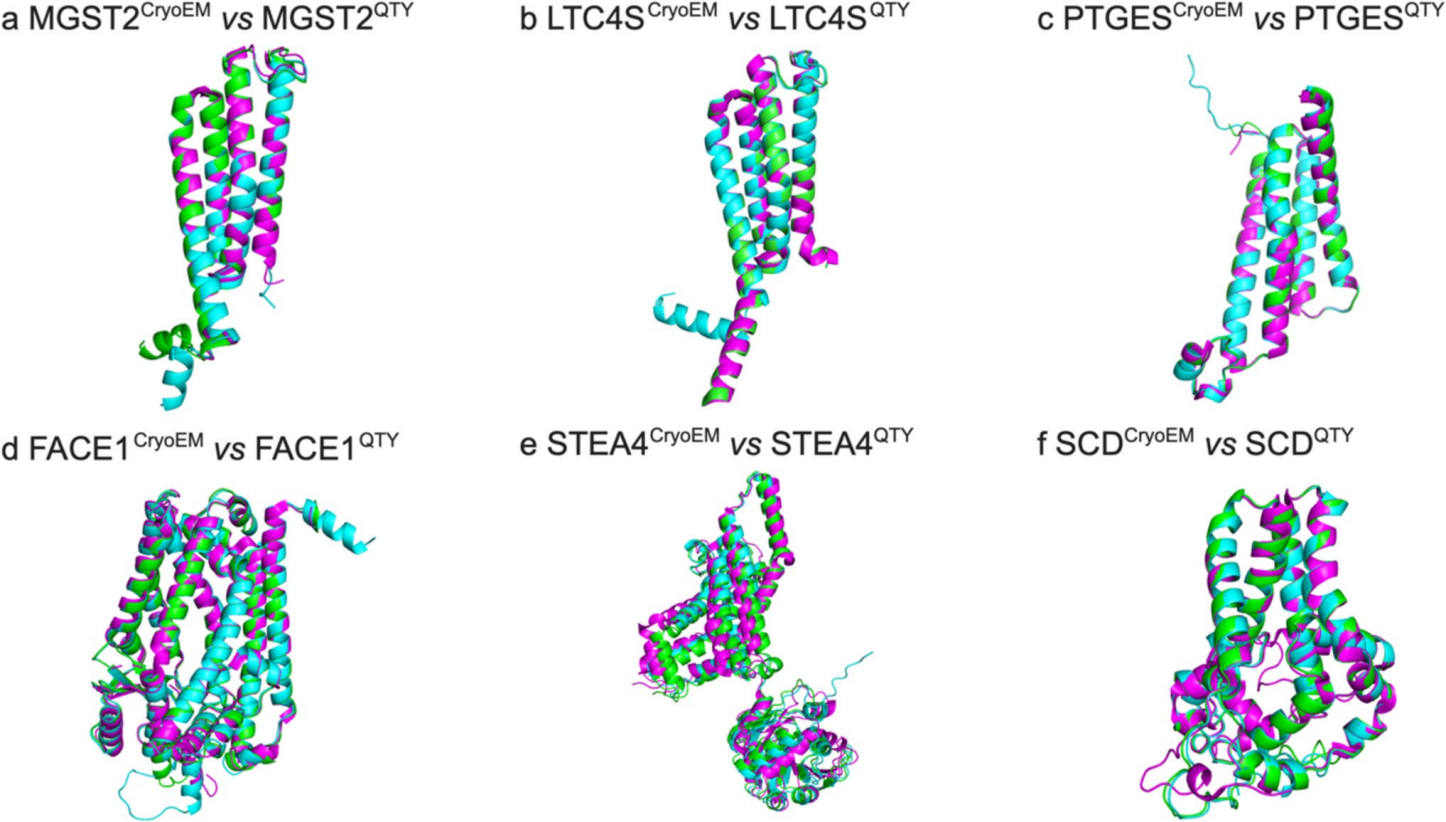
Superpositions of CryoEM structures with AlphaFold3-predicted native integral membrane enzymes and their water-soluble QTY analogs. Superposition of i) the experimentally determined CryoEM structures (magenta) with ii) AlphaFold3-predicted structures (green) and iii) AlphaFold3-predicted water-soluble QTY variant structures (cyan). These superpositions are shown in Fig. 4. These three different kinds of structures are apparently superposed very well. The differences and variations are insignificant. (**a**) MGST2^CryoEM^/MGST2^Native^/MGST2^QTY^, (**b**) LTC4S^CryoEM^/LTC4S^Native^/LTC4S^QTY^, (**c**) PTGES^CryoEM^/PTGES^Native^/PTGES^QTY^, (**d**) FACE1^CryoEM^/FACE1^Native^/FACE1^QTY^, (**e**) STEA4^CryoEM^/STEA4^Native^/STEA4^QTY^, (**f**) SCD^CryoEM^/SCD^Native^/SCD^QTY^.

We also superposed the CryoEM structures, AlphaFold3-predicted native structures, and AlphaFold3-predicted QTY analogs. The three different kinds of structures superposed very well (Fig. 5). They not only validates the high level of similarity between experimentally determined structures and their QTY analogs, but also between AlphaFold3-predicted native structures and AlphaFold3-predicted QTY analogs (Figs. 1 and 3), we also assessed how the experimentally determined Cryo-EM structures compare with AlphaFold3-predicted native structures. The close superpositions of AlphaFold3-predicted native structures with experimentally determined structures confirms the accuracy of AlphaFold3. This suggests that water-soluble QTY analog integral transmembrane enzymes could potentially be expressed, purified, and used as soluble antigens for generating therapeutic monoclonal antibodies.

### Analysis of the Hydrophobic Surface of Native Membrane Enzymes and their Water-Soluble QTY Analogs

The six integral transmembrane enzymes that we selected are hydrophobic and water-insoluble. For example, the MAPEG family proteins show high hydrophobicity in sequence position 80–120 with 3–4 membrane spanning segments. To effectively study these membrane enzymes, they must first be isolated from their lipid bilayer membranes. This process requires detergents, which solubilize the transmembrane proteins by disrupting the hydrophobic interactions between the membrane enzymes and the lipid bilayer. Without screening and optimizing the appropriate detergents, these membrane enzymes aggregate and precipitate upon isolation, resulting to a loss of their biological functions.

**Fig. 6:**
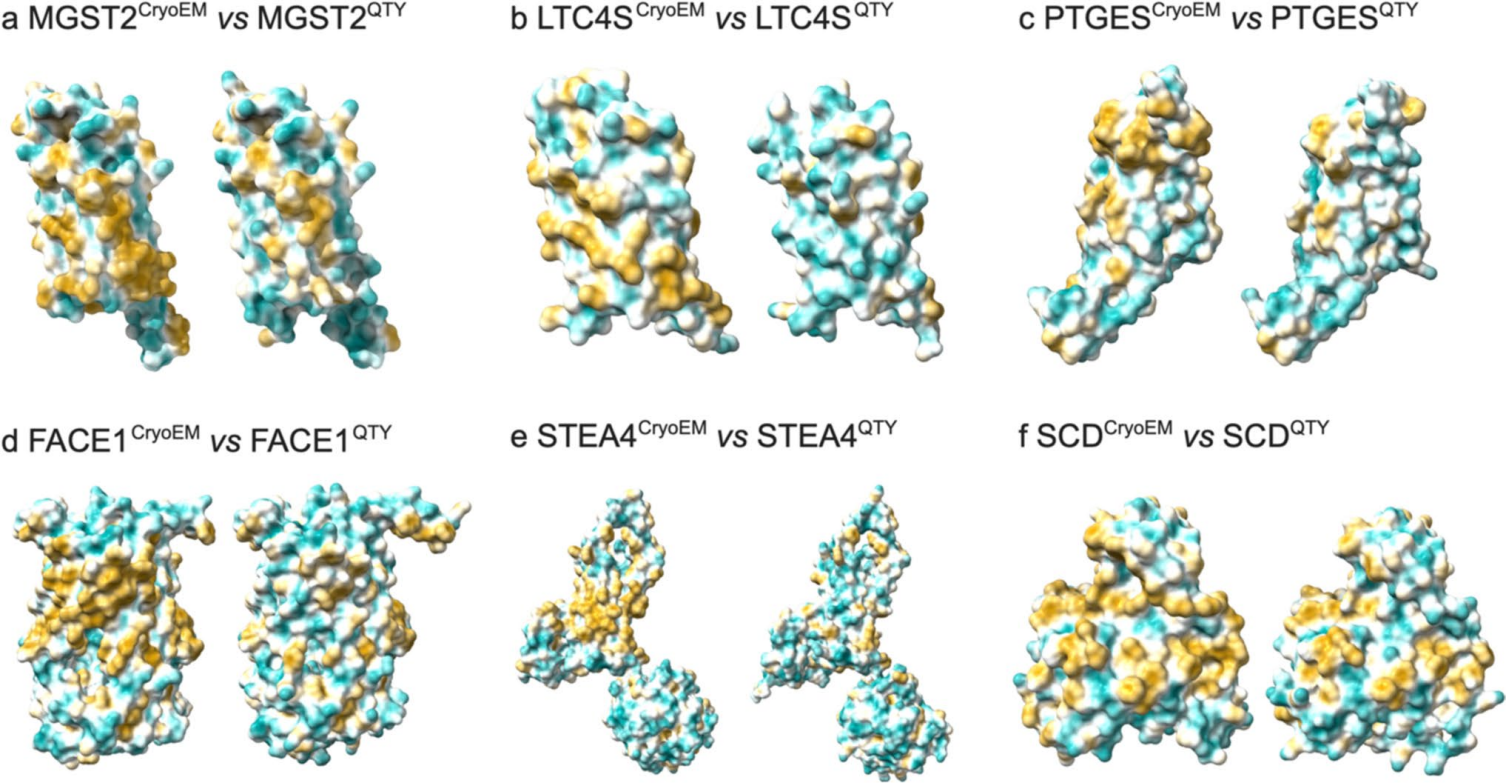
Hydrophobic surface of six integral membrane enzymes and their water-soluble QTY analogs. The native proteins have many hydrophobic residues L, I, V, and F in the transmembrane helices. After Q, T, and Y substitutions of L, I and V, and F respectively, the hydrophobic surface patches (yellowish) in the transmembrane helices become more hydrophilic (cyan). For clarity of direct comparisons, unstructured loops in the CryoEM structures were removed in the QTY analogs. (**a**) MGST2 *vs* MGST2^QTY^, (**b**) LTC4S *vs* LTC4S^QTY^, (**c**) PTGES *vs* PTGES^QTY^, (**d**) FACE1 *vs* FACE1^QTY^, (**e**) STEA4 *vs* STEA4^QTY^, and (**f**) SCD *vs* SCD^QTY^.

The yellowish hydrophobic surface areas (Fig. 6) originate from the transmembrane domains of the enzymes, which are embedded within the hydrophobic lipid bilayer. In these regions, nonpolar and hydrophobic Leucine (L), Isoleucine (I), Valine (V), Phenylalanine (F), Methionine (M), Tryptophan (W), and Alanine (A) interact with lipid molecules to exclude water.

By applying the QTY code to systematically replacing the hydrophobic amino acids L, I/V, F, with hydrophilic amino acids glutamine (Q), threonine (T), and tyrosine (Y), the hydrophobic surfaces are significantly reduced. Furthermore, the QTY substitutions do not significantly alter the alpha-helix [37] structure of these analogs. This observation is consistent with previous experiments showing that QTY analogs of chemokine and cytokine receptors retain their structural integrity, stability, and ligand-binding activity, after becoming water-soluble [37–40].

### Analysis of the Substrate Binding Ability of Native Membrane Enzymes and the Water-Soluble QTY Analogs

**Fig. 7:**
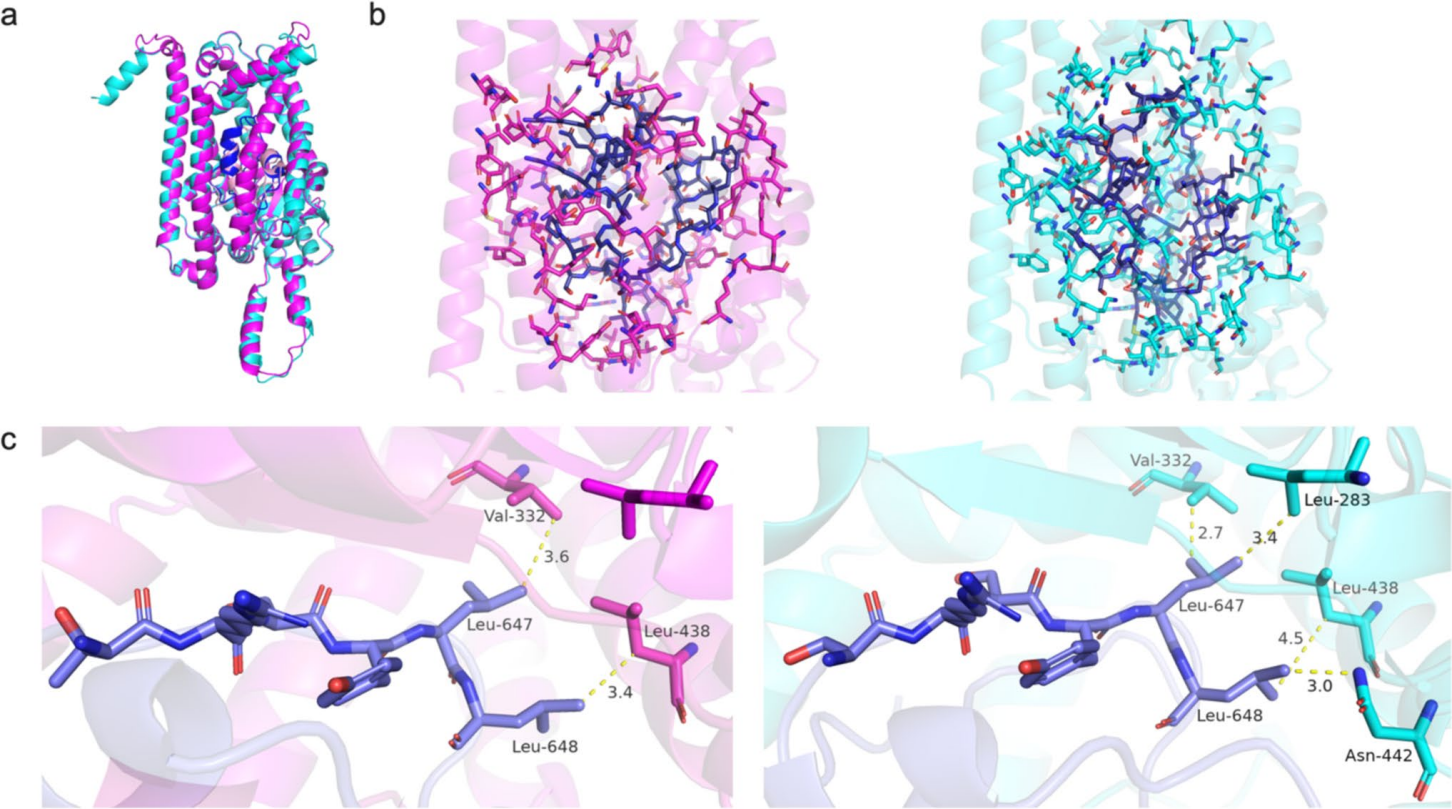
Binding site analysis for FACE1 and Prelamin A. Color code: magenta = CryoEM determined FACE1 structure; cyan = AlphaFold3-predicted water-soluble FACE1; pink = CryoEM determined Prelamin A structure; blue = AlphaFold3-predicted Prelamin A; light blue = Prelamin A residues. (**a**) Superposition of the active site of AlphaFold3-predicted CryoEM determined FACE1 binding with Prelamin A and AlphaFold3-predicted QTY variant of FACE1^QTY^ binding with Prelamin A. The structures superposed well, with RMSD of 0.469 Å; (**b**) The side-by-side comparison of active site in CryoEM determined FACE1 binding with Prelamin A and QTY variant of FACE1^QTY^ binding with Prelamin A; (**c**) Side-by-side comparison of detailed molecular interaction.

We first used AlphaFold3 to predict the enzyme binding ability of FACE1 with Prelamin A and the substrate binding ability of STEA4 with FAD (Flavin-adenine dinucleotide) and NADPH (Nicotinamide adenine dinucleotide phosphate). Following these predictions, we superposed the native structures with their corresponding QTY analogs to evaluate whether the QTY analogs retained their binding capabilities (Fig. 7).

Our analysis revealed that the structures aligned well, suggesting that the QTY analogs of both FACE1 and STEA4 maintained their protein binding stability and structural integrity. The results also align with previous studies, where the C-terminal part of prelamin A must enter the chamber formed by the seven transmembrane spans of FACE1 [20]. We also see that prelamin A is proteolytically cleaved between residues Y646 and L647 by ZMPSTE24 to produce mature lamin A. In addition, we note that prelamin (~ 74 kD) is too large to fit entirely within the catalytic chamber, where the C-terminal domain of prelamin A is threaded into the chamber for cleavage [23]. In both cases, the first binding pocket composed of Val332 and Leu283 permitted the entry of Leu647 (Fig. 7c). However, the second binding pocket of the QTY variant showed Leu648 interacting with Asn442 instead of Leu438, which could indicate that the hydrophobic nature of the binding pocket encouraged the hydrogen bonding with Asn442 nearby.

**Fig. 8:**
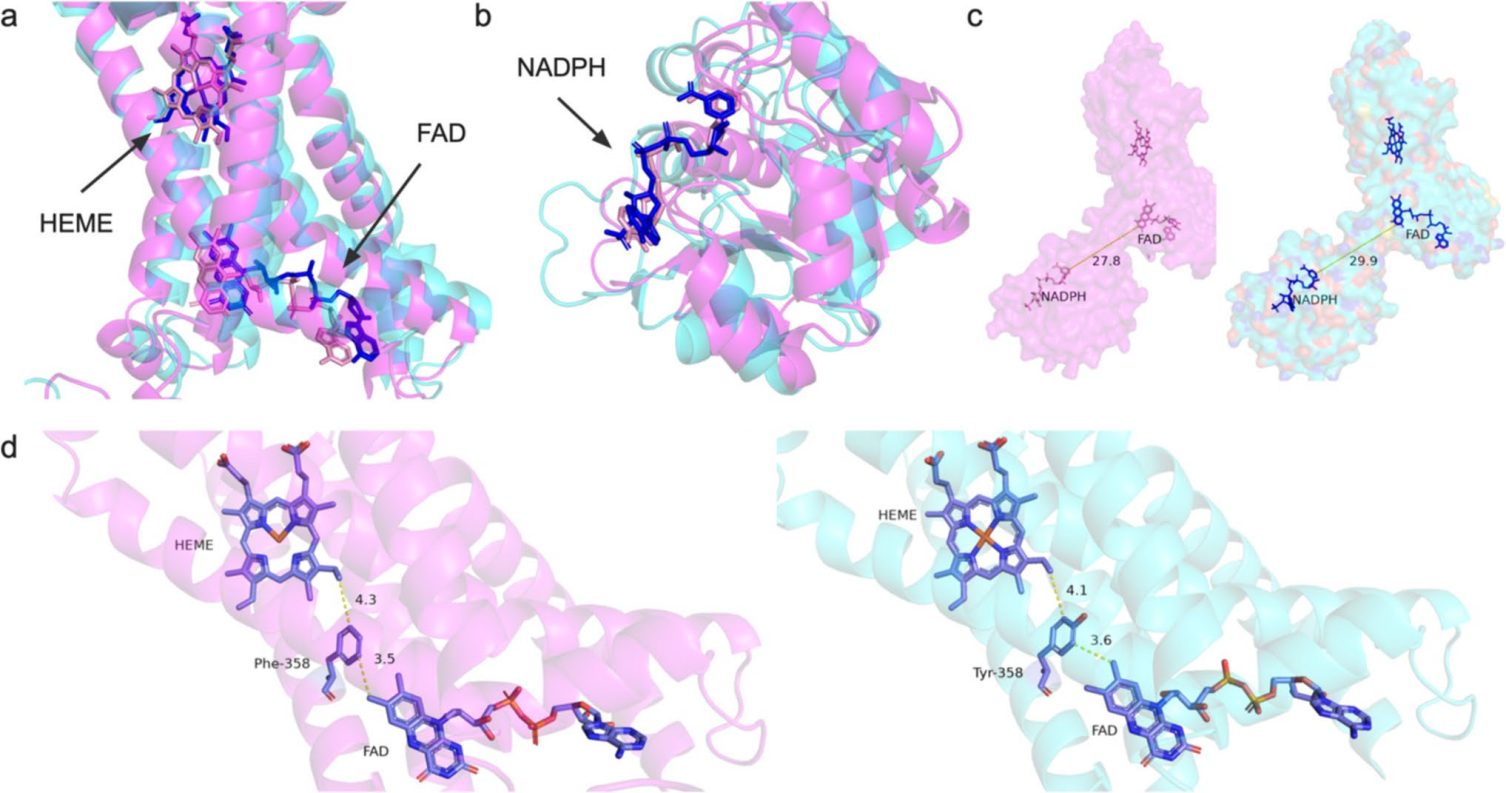
Binding site analysis for integral membrane enzyme STEA4 and heme, FAD, and NADPH. Color code: magenta = CryoEM determined STEA4 structure; cyan = AlphaFold3-predicted water-soluble STEA4; magenta = CryoEM determined ligands structure; blue = AlphaFold3-predicted ligands structure; blue = substrates. (**a**) Superposition of the active site of AlphaFold3-predicted CryoEM determined STEA4 binding with heme and FAD and AlphaFold3-predicted QTY variant of STEA4^QTY^ binding with heme and FAD. The structures superposed well; (**b**) Superposition of the active site of AlphaFold3-predicted CryoEM determined STEA4 binding with NADPH and AlphaFold3-predicted QTY variant of STEA4^QTY^ binding with NADPH. The structures superposed well; (**c**) The side-by-side comparison of the cavity between FAD and NADPH in STEA4 CryoEM structure and QTY analog STEA4^QTY^; (**d**) The side-by-side comparison of active site in CryoEM determined STEA4 binding with heme and FAD, as well as the motif formed with Phe358, which allows for electron transport.

The STEA4 oxidoreductase draws two electrons from NADPH before passing the electrons to FAD at the cytosolic face of the transmembrane domain [27], where alpha-helices H2, H3, H4, and H5 forms the cofactor-binding core containing FAD with Heme. We note that the distance between the FAD and Heme is 9.3 Å. and are both within 4 Å of residue F359, which enables electron to travel through the FAD-F359- Heme motif. NADPH resides in an open and solvent-exposed pocket and is bound in a similar fashion with a stacked flavin-like F420 molecule, suggesting potential sites where FAD can be stacked similarly [25, 56]. The cavity between FAD and NADPH shows putative tunnel for FAD transport without requiring conformational protein rearrangements (Fig. 8).

### AlphaFold3 Predictions

We experiment with DeepMind’s latest model, AlphaFold3, which was released in May 2024. Unlike AlphaFold2 and other physics-based prediction models, AlphaFold3 offers a unified deep-learning framework capable of predicting a wide range of biomolecules, including ligands, ions, nucleic acids, and modified residues [49, 50]. Despite its generalized approach, AlphaFold3 outperforms specialized methods in all but one category, and is reported to be 50% more accurate than physics-based tools on PoseBusters benchmark [49, 50].

Moreover, with the introduction of an online server, AlphaFold3 is now easily accessible, allowing each user to generate up to 20 predictions per day. The structure predictions of the QTY analogs were performed using the AlphaFold3 [49] server, which was accessed at (https://alphafoldserver.com). The predictions was run online, free of charge, following the instructions provided on the website.

DeepMind also collaborates with the European Bioinformatics Institute (EBI) to make over 214 million predicted protein structures available through the AlphaFold Protein Structure Database (https://alphafold.ebi.ac.uk) [53]. This number is expected to grow over time, with the quality of predictions improving further with the advancements in AlphaFold3.

While AlphaFold3 represents a significant advancement, offering valuable insights and a strong starting point, it still has some limitations. For example, the pLDDT scores demonstrated a high level of confidence in the accuracy of the predictions, particularly in the helical regions. However, it is important to consider that pLDDT scores are based on the training data, which may not fully capture certain biological contexts [51]. In addition, AlphaFold3 suffers from hallucination, where the model might predict plausible but non-existent molecular structures [49, 50]. AlphaFold3 is also limited in protein-ligand predictions, with only a fixed number of ligands to choose from. Therefore, it is best used as an initial guide, with further rigorous experimental validation needed to confirm and refine its predictions.

### The Integral Transmembrane Enzymes in this Study

We investigate the application of the QTY code across a broader range of integral membrane enzymes and assess whether QTY analogs retain their substrate binding abilities and later to experimentally study their enzymatic activities. The proteins selected for this study—MGST2, LTC4S, PTGES, FACE1, STEA4, and SCD—are known for their significant biological and medical relevance. These membrane enzymes were chosen due to their diverse roles in critical cellular processes and their potential as therapeutic targets. We believe these integral membrane enzymes will help us advance our understanding of the QTY code’s efficacy and its applications for protein engineering.

The water-soluble QTY analogs of the proteins in this study have several potential applications: **i**) they could help generalize the QTY code system across a broader range of integral membrane protein families, **ii**) they may serve as soluble antigens for discovery of therapeutic monoclonal antibodies for metabolic disorders and cancer, and **iii**) the purified soluble integral membrane enzymes could be used in high-throughput drug discovery efforts.

## Conclusion

Proteins can generally be divided into two classes: Class I is hydrophilic and Class II is hydrophobic [37, 57, 58]. More specifically, each class proteins are composed of three chemically distinct but structurally similar of alpha-helix: **i**) type I alpha-helix is comprised of hydrophilic amino acids (D, E, N, Q, H, K, R, S, T and Y) commonly found in water-soluble globular proteins, **ii**) type II alpha-helix comprised of hydrophobic amino acids (L, I, V, F, M, A, W and P) and is commonly found in the alpha-helical transmembrane segments of membrane proteins, **iii**) type III alpha-helix amphiphilic proteins are composed of nearly equal amounts of hydrophilic and hydrophobic amino acids, which are often partitioned into distinct hydrophobic and hydrophilic faces [37, 57, 58].

In our current study, we applied QTY code to six hydrophobic integral transmembrane enzymes and convert them into water-soluble QTY analogs. To compare the QTY analogs with their native protein structures, we first used AlphaFold3 to predict the structures of the QTY analogs, and then superposed them onto their respective native enzyme structures. We also used a range of in silico computational and bioinformatic tools to analyze the sequence and structural characteristics related to protein stability and water solubility. With the help of AlphaFold 3, we were able to show that FACE1 and STEA4 maintained their substrate binding ability despite the QTY substitutions.

In the future, we hope to investigate whether the QTY mutations are safe in preserving the native enzyme mechanisms. Replacing Phenylalanine (F) with redox active amino acids such as Tyrosine (Y) in electron transfer and proton-coupled electron transfer could potentially enhance the electron transfer efficiency [59]. On the other hand, studies have found that hydrophobic environments in proteins increases the reduction potential of metal centers by disfavoring high redox states [60]. Thus, using QTY code to reduce the hydrophobicity of proteins could lead to decrease in redox potential. The overall redox potential change is a complicated analysis that we hope to address in future experiments.

Our findings revealed that 1) the native proteins and their QTY analogs maintained high levels of structural similarity. 2) The QTY code effectively reduced the hydrophobic surfaces of the membrane enzymes. This suggests that the QTY enzyme analogs likely retain their original functions. Therefore, these hydrophilic QTY enzymes hold significant potential to use as soluble antigens for therapeutic monoclonal antibody discovery for the treatment of cancer and other diverse diseases.

## Method

### Protein Sequence Alignments and Other Characteristics

The native protein sequences for MGST2, LTC4S, PTGES, FACE1, STEA4, and SCD were obtained from UniProt (https://www.uniprot.org). The sequences for the QTY analogs were aligned using the same methods as previously described. The MWs and pI values of the proteins were calculated using the Expasy (https://web.expasy.org/compute_pi/).

### AlphaFold3 Predictions

The protein structures of the QTY analogs were predicted using AlphaFold3 server (https://alphafoldserver.com/). PBD files for the predicted native protein structures were obtained from The EBI (https://alphafold.ebi.ac.uk), which contains all AlphaFold3 predicted structures for native proteins. The UniProt website (https://www.uniprot.org) provided protein ID, entry name, description, and FASTA sequence for each native protein. The QTY code can be applied to FASTA sequences through the QTY method website (https://pss.sjtu.edu.cn/). The website also provides MWs, pI values, TM variation, and overall variation.

### Superposed Structures

PBD files for native protein structures experimentally determined by CryoEM were taken from the PDB include MGST2 (PDB: 6SSS), LTC4S (PDB: 3PCV), PTGES (PDB: 4AL0), FACE1 (PDB: 5SYT), STEA4 (PDB: 6HCY), and SCD (PDB: 4ZYO). Predictions for the QTY analogs were carried out using the AlphaFold3 server, which can be found at https://alphafoldserver.com/. These structures were superposed and the RMSDs were calculated using PyMOL (https://pymol.org). For simplicity and clarity, unstructured loops and extraneous protein monomers were removed from the figures.

### Structure Visualization

PyMOL (https://pymol.org) was used to superpose the native protein structure and the QTY variant. UCSF Chimera (https://www.rbvi.ucsf.edu/chimera) was used to render each protein model with hydrophobicity patches.

### Data Availability of AlphaFold3-Predicted Water-Soluble QTY Analogs

European Bioinformatics Institute (EBI, https://alphafold.ebi.ac.uk) serves as a database that provides open access to more than 214 million AlphaFold3-predicted protein structures. The QTY code designed water-soluble analogs of the human integral membrane protein enzymes are available at https://github.com/EdwardChen777/membrane_enzymes. If additional information is needed, please contact Edward Chen, edwardchen5414@gmail.com. Protein characteristics used in the analysis are available on UniProt (https://www.uniprot.org/). The native CryoEM-determined six integral membrane protein enzymes are available in the RCSB PDB repository (https://www.rcsb.org/).

## Data Availability

Each statistical and computational analysis of this study, included with step-by-step instructions where possible, are publicly available to ensure repeatability. For more detailed information on the statistical analyses, input files and detailed outputs, including the AlphaFold2 calculations and codes to regenerate analyses, please visit the website: https://github.com/karagol-alper/QTY-dynamics-EAA13. Further information and requests for data should be directed to and will be fulfilled by A.K. alper.karagol@gmail.com, and T.K. taner.karagol@gmail.com.
